# The Role of Early Life Experience and Species Differences in Alcohol Intake in Microtine Rodents

**DOI:** 10.1371/journal.pone.0039753

**Published:** 2012-06-22

**Authors:** Allison M. J. Anacker, Todd H. Ahern, Larry J. Young, Andrey E. Ryabinin

**Affiliations:** 1 Department of Behavioral Neuroscience, Oregon Health & Science University, Portland, Oregon, United States of America; 2 Center for Behavioral Neuroscience, Department of Psychology, Quinnipiac University, Hamden, Connecticut, United States of America; 3 Center for Translational Social Neuroscience, Department of Psychiatry and Behavioral Sciences, Yerkes National Primate Research Center, Emory University, Atlanta, Georgia, United States of America; University of Western Ontario, Canada

## Abstract

Social relationships have important effects on alcohol drinking. There are conflicting reports, however, about whether early-life family structure plays an important role in moderating alcohol use in humans. We have previously modeled social facilitation of alcohol drinking in peers in socially monogamous prairie voles. We have also modeled the effects of family structure on the development of adult social and emotional behaviors. Here we assessed whether alcohol intake would differ in prairie voles reared by both parents compared to those reared by a single mother. We also assessed whether meadow voles, a closely related species that do not form lasting reproductive partnerships, would differ in alcohol drinking or in the effect of social influence on drinking. Prairie voles were reared either bi-parentally (BP) or by a single mother (SM). BP- and SM-reared adult prairie voles and BP-reared adult meadow voles were given limited access to a choice between alcohol (10%) and water over four days and assessed for drinking behavior in social and non-social drinking environments. While alcohol preference was not different between species, meadow voles drank significantly lower doses than prairie voles. Meadow voles also had significantly higher blood ethanol concentrations than prairie voles after receiving the same dose, suggesting differences in ethanol metabolism. Both species, regardless of rearing condition, consumed more alcohol in the social drinking condition than the non-social condition. Early life family structure did not significantly affect any measure. Greater drinking in the social condition indicates that alcohol intake is influenced similarly in both species by the presence of a peer. While the ability of prairie voles to model humans may be limited, the lack of differences in alcohol drinking in BP- and SM-reared prairie voles lends biological support to human studies demonstrating no effect of single-parenting on alcohol abuse.

## Introduction

Social relationships and alcohol drinking interact in complex ways. Social relationships can facilitate or inhibit alcohol drinking, and alcohol consumption can markedly influence social relationships. Interactions between alcohol, other drugs of abuse, and social behaviors, along with a deeper understanding of neural reward mechanisms, have led to the prominent hypothesis that the neural circuits underlying the reinforcing properties of alcohol and other addictive drugs overlap with circuits underlying natural rewards, including social attachment [1], [2], [3], [4], [5], [6], [7].

Prairie voles (*Microtus ochrogaster*) are socially monogamous rodents that have been studied for their unusual social behaviors in the field and in the laboratory. Like humans, they form long-term pair bonds with mates, spend much of their time together, and both parents typically participate in the care of offspring [8], [9], [10], [11], [12]. In addition, over two-thirds of prairie voles do not disperse from their natal nests and instead help rear future litters [13], [14]. There is an extensive literature characterizing the neural circuits that drive and regulate social behaviors in prairie voles. Based on this, we previously established prairie voles as an animal model to study the effects of social relationships on alcohol intake. Specifically, we showed that prairie voles drink more alcohol when introduced to alcohol with a sibling than when isolated [15], whereas mice and rats typically drink more in isolation (reviewed in [16]). We have also demonstrated that same-sex non-sibling peers can have a direct impact on altering the level of alcohol consumption in this species [17].

Meadow voles (*Microtus pennsylvanicus*) are closely related to prairie voles, but exhibit very different social behaviors. They do not form lasting reproductive partnerships, and in summer months do not appear to spend time with other conspecifics for purposes other than breeding [18], [19]. In winter months, however, meadow voles become more social, often living in communal groups [20], [21], [22]. When tested in the laboratory, males show a preferential bond for a same-sex sibling under both winter- or summer-like light conditions; however, females show a same-sex partner preference for a sibling or cagemate only under non-breeding, winter-like conditions [23], [24], [25]. Compared to prairie voles, meadow voles exhibit a number of differences in the social reward circuitry, which contribute to the observed differences in bonding behavior. For example, compared to prairie voles, meadow voles have lower densities of vasopressin 1a receptors (V1aR) and oxytocin receptors (OTR) in the ventral pallidum and nucleus accumbens, respectively [26], [27]. These are reward-related structures that have been implicated in alcohol and drug abuse. Further, experimentally increasing V1aR or OTR densities in these regions enhances social bond formation [28], [29], [30], [31]. In light of the differences in social reward circuitry, we tested the hypothesis that meadow voles would differ from prairie voles in their alcohol drinking, particularly within a social context.

In addition to species differences, we also aimed to examine whether early life family structure could impact adult alcohol drinking behaviors. In humans, parenting is thought to be an important influence on a variety of offspring behaviors, including use and abuse of alcohol and other drugs. However, the literature comparing single-parent and two-parent homes is mixed. Some studies, including a meta-analysis [32], indicate that children from single-parent homes show a greater propensity to use and abuse alcohol [33], [34], [35], [36], [37], while others do not, or show that the effects are weaker than other mediators [38], [39], [40], [41]. Prairie voles offer a unique opportunity to experimentally test the connection between early family structure and adult social and nonsocial alcohol consumption. Not only have we shown that prairie voles exhibit socially-moderated drinking behavior [15], [17], we have also demonstrated that prairie voles reared by single-mothers (SM) exhibit decreased prosocial behaviors in adulthood compared to offspring reared biparentally (BP) [42], as have others in monogamous mandarin voles [43], [44].

Here we tested the hypothesis that SM-reared prairie voles would consume more alcohol and be less influenced by social conditions than BP-reared prairie voles. To our knowledge, this is the first experimental assessment of the influence of early life family structure on adult drinking under different social conditions.

## Methods

### Experiment 1

#### Animals

Adult prairie voles and meadow voles were bred and housed in the colonies at Emory University where this experiment took place. Due to the limited availability of meadow voles, only male subjects were used. All animals received food (Lab Diet, rabbit chow) and water *ad libitum* throughout the experiments, and were kept on a 14∶10 light dark cycle, analogous to the breeding season.

The prairie voles consisted of animals from two different rearing conditions, identical to those described by Ahern and Young [42], [45]. Briefly, 18 days after breeders were paired, the male was removed from cages randomly assigned to the single mother (SM) condition; males assigned to the biparental (BP) condition remained partnered throughout the study. Pups from both rearing conditions were born 24–28 days post-pairing and were weaned at 21 days of age and housed in same-sex pairs of the same rearing condition. SM-reared prairie voles (n = 10, 1–3 pups used from each of 6 litters) were 81–82 days old, and BP-reared prairie voles (n = 12, 2 pups used from each of 6 litters) were 80–82 days old at the start of the experiment.

Meadow voles (n = 12, 1–3 pups used from each of 6 litters) were reared biparentally, weaned at 21 days of age and housed in same-sex pairs. Again, due to limited meadow vole availability, older meadow voles were used for this study, ranging from 95–174 days (mean ± SEM: 138±8.68). While prairie voles and meadow voles differed in age in Experiment 1, we directly assessed the effect of age on alcohol drinking in Experiment 2.

All experiments were approved by the Emory Institutional Animal Care and Use Committee.

#### Apparatus and recording

**Figure 1:**
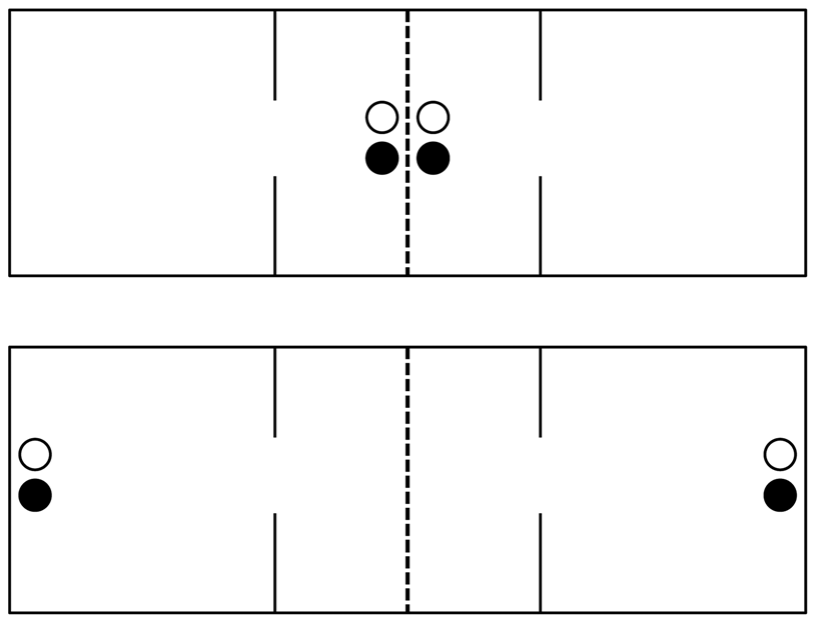
Drinking apparatus. ‘Bird’s eye view' schematic of the drinking apparatus in the social drinking condition (top) and non-social drinking condition (bottom). Solid lines indicate cage walls, dashed lines indicate the wire divider, and circles indicate drinking tubes. Clear circles represent water; black circles represent 10% alcohol in water.

The behavioral observation apparatus, recording equipment, and behavior tracking software have been described in detail [46]. We adapted the cages previously used for partner preference testing to be used for drinking experiments as follows. The large three-chamber cages (Fig. 1) were divided in half by placing a wire mesh divider similar to that described by Anacker et al. [15] in the center of the middle chamber. Each animal was thereby restricted to its own half of the cage and had exclusive access to a set of drinking tubes, but had visual, olfactory, auditory, and limited tactile contact with its cagemate. We have previously validated the use of mesh dividers as a way to detect individual fluid intake levels, observing similar drinking patterns in these conditions compared to pair-housed animals without dividers [15]. One member of each pair was spot-shaved on the dorsal side for identification purposes prior to the start of the experiment, so that each animal was placed in the same side of the apparatus each day.

Drinking solutions (described below) were made available to each animal in two drinking tubes, consisting of a 25-ml glass graduated cylinder fitted with a rubber stopper and metal sipper tube, as described in Anacker et al. [15]. These tubes were either placed on the wire divider in the center of the cage such that each animal could drink while near the other (social drinking condition, SD) or placed on opposite ends of the apparatus such that the animals would not be near each other while drinking (non-social drinking condition, NSD) as shown in Fig. 1.

### Solutions

Alcohol (190 proof ethanol) was available at 10% concentration by volume, diluted in tap water. Tastants (saccharin and quinine) were also separately diluted in tap water (0.05% and 0.0025% by weight, respectively).

Ethanol injections were given at a concentration of 20% by volume in saline. Each animal received an alcohol dose of 2.5 grams per kilogram body weight, resulting in a range of volume injected per animal of 0.42–0.95 mL.

#### Procedure

Cagemates were taken from their home cage and placed in the alcohol drinking apparatus described above for four hours per day, with one vole on each side of the divider. The first five days (days 1–5) were for acclimation to the cage and drinking tube placement, with water present in all drinking tubes. The following four days (days 6–9), each animal had access to a tube with water and a tube with 10% alcohol. The 4-hour drinking session was split into two 2-hour sessions, one SD and one NSD. The order of SD and NSD sessions was counterbalanced across pairs. The sessions began at the onset of the light phase, at which time we have previously observed a slight peak in alcohol intake [15].

For each animal, fluid levels were measured before and after each 2-hour session, and these levels were used to calculate alcohol preference and alcohol dose consumption per body weight. Alcohol preference was calculated by taking the volume of alcohol consumed and dividing by total fluid consumption (water and alcohol). The dose of alcohol consumed was calculated as grams of alcohol consumed per kilogram body weight (g/kg). Four tubes containing drinking solutions were positioned in an identical manner on an empty cage in order to detect changes in fluid levels due to leakage or other disturbance. No change was detected in the volume of these control tubes during the sessions and so no adjustment was made to the volumes consumed by the voles. Further, all drinking sessions were digitally recorded and behavior was tracked for a subset of animals (n = 8 per group) using Social Scan 2.0 (CleverSys Inc.).

To assess potential differences in taste between groups, all animals were again placed in the testing chambers and given access to water and saccharin on day 10, and water and quinine on day 11. On both days, animals had access for two hours under the NSD condition, since we have previously shown no effect of social housing on tastant consumption [15], [17].

After the final self-administration session, cagemates were returned to their home cage and left undisturbed until day 16. On day 16, one week following alcohol access, all animals were injected intraperitoneally with 2.5 g/kg ethanol, placed back into home cages, and 90 minutes later euthanized by CO_2_ inhalation followed by decapitation. Trunk blood was taken to determine blood ethanol concentration (BEC) and examine potential differences in ethanol elimination rates. Sera were frozen and shipped on ice to the laboratory at Oregon Health & Science University, where BECs were determined using an Analox Analyzer (Analox Instruments, Luneburg, MO, USA). One prairie vole exhibited an extremely low BEC similar to that expected from an animal without an injection. This animal was removed from analysis of BEC.

#### Statistical Analyses

For the behavioral tests, the BP group (which represents how prairie voles are typically reared in the laboratory) was designed to be the control group for both the SM prairie voles and the meadow voles. However, since we observed no differences on any measure between SM- and BP-reared prairie voles, as discussed below, we combined all prairie voles into one group and compared them to the meadow voles.

We compared the overall preference and the dose of alcohol consumed in the SD and NSD periods between species. Each dependent variable (i.e. preference and dose) was averaged across all four days since no within-group differences in drinking patterns between days were observed. Drinking data were compared by repeated measures ANOVA, with species as the between-subjects factor, drinking condition (SD or NSD) as the repeated measure, and preference or dose as the dependent variable. There was no effect of the order of drinking condition on the dependent variables, and so the order is not presented.

We also compared saccharin and quinine preference and doses, the average distance traveled as reported by the Social Scan software, BECs following injection, and age and body mass differences between species. Group differences were analyzed by t-tests, with Welch's correction for unequal variances where appropriate; corrected values are reported.

### Experiment 2

#### Animals

Animals used in this study were prairie voles bred at the Portland Veterans Affairs Medical Center, Veterinary Medical Unit, where this experiment took place. Food (LabDiet Hi-Fiber Rabbit chow, Nutrena Cleaned Grains corn, and Grainland Select Grains oats) and water were available *ad libitum*. These prairie voles were young adults (78–91 days; n = 8, 1–3 pups used from each of 4 litters) or older adults (165–167 days; n = 10; 1–4 pups used from each of 4 litters) to match the age (and thus weight) of the prairie and meadow voles, respectively, in Experiment 1.

This experiment was approved by the Portland Veterans Affairs Medical Center Institutional Animal Care and Use Committee.

#### Procedure

The goal of the experiment was to examine the effect of age and body size on alcohol elimination. Because prior exposure to alcohol can affect subsequent metabolism of alcohol [15], the animals in Experiment 2 were allowed to self-administer alcohol prior to ethanol injections and BEC analysis. Paralleling Experiment 1, prairie voles had four days of 4-hour access to 10% ethanol and water, starting just after the onset of the light phase. In this case however, drinking took place in the home cage, in which the animals were separated by a divider as described previously [15], and the drinking tubes were always available at the divider, similar to the SD condition in Experiment 1. Fluid levels were checked before and after each 4-hour session, and were used to calculate alcohol preference and the dose of alcohol consumed in grams per kilogram body weight (g/kg).

One week after alcohol drinking, all animals were injected intraperitoneally with 2.5 g/kg ethanol and, 90 minutes later, euthanized by CO_2_ inhalation followed by decapitation with trunk blood taken to determine BEC as in Experiment 1.

## Results

**Figure 2:**
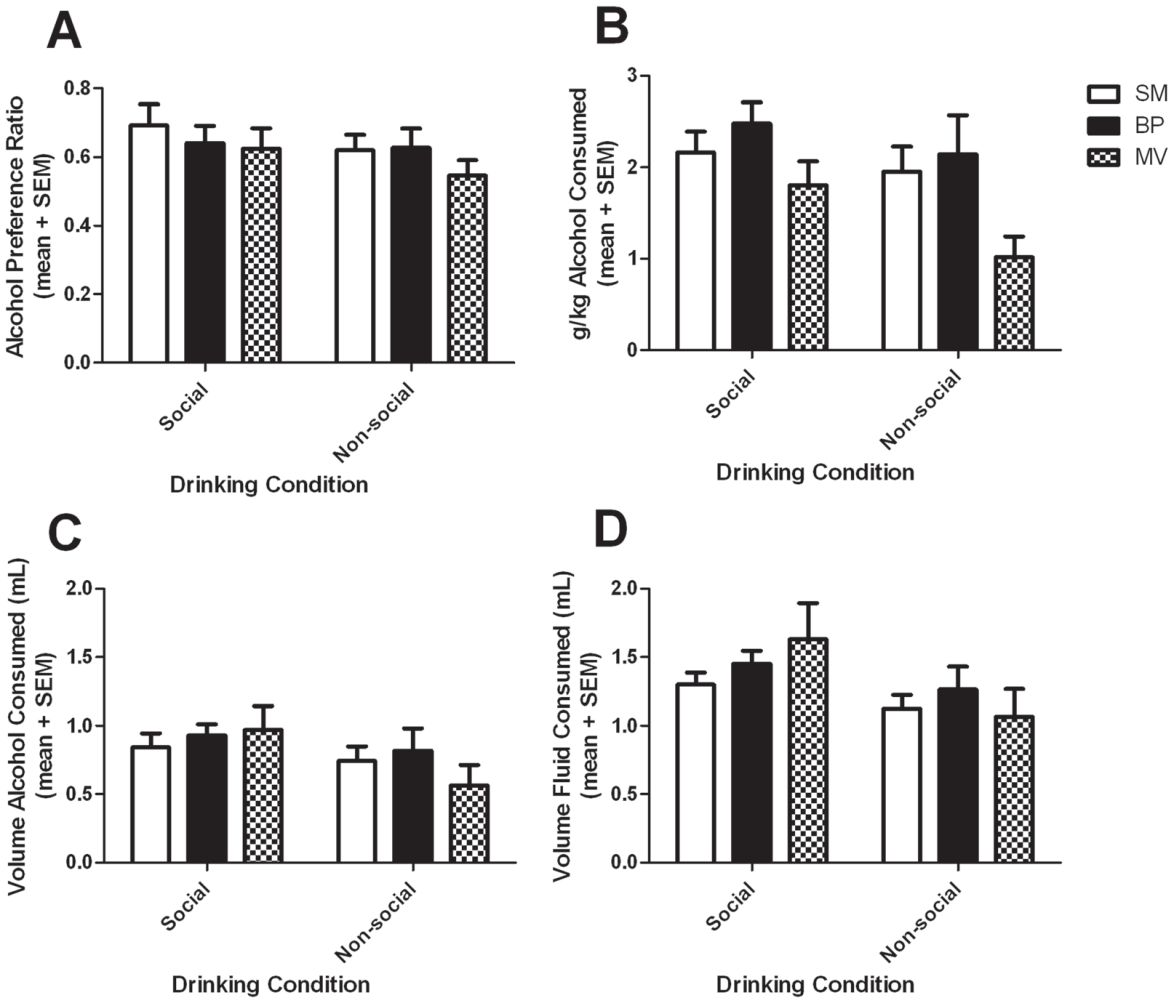
Alcohol drinking in 2-hour social and non-social drinking conditions. There was no difference between rearing group, species or drinking condition in alcohol preference (A). There was no significant difference between rearing groups in alcohol consumption, but there was a significant difference between species (B), where meadow voles consumed less alcohol than prairie voles (p<0.05). There was a significant effect of drinking condition on alcohol consumption, where animals consumed less in the non-social condition than the social condition (p<0.005). There was no interaction between group or species and drinking condition. There was no significant difference between rearing group or species on alcohol volume consumed (C) or total fluid volume consumed (D), but there was an effect of drinking condition where less was consumed during the non-social period than in the social period (p<0.05). Values indicate group mean + standard error of the mean (SEM). Single mother-reared prairie voles, SM; biparentally-reared prairie voles, BP; meadow voles, MV.

In Experiment 1, prairie voles and meadow voles had access to 10% alcohol and water in social drinking (SD) and non-social drinking (NSD) conditions. There was no difference in alcohol preference or the dose consumed per body weight between SM- and BP-reared prairie voles (Fig. 2), and so they were combined into one group for comparison with meadow voles. There was no main effect of species on preference for alcohol over water (F(1,32) = 1.11; p = 0.30), but a trend toward an effect of social condition where alcohol preference was lower in the NSD than the SD condition (F(1,32) = 3.47; p = 0.07); there was no interaction between species and drinking condition (F(1,32) = 0.38; p = 0.54; Fig. 2A). There was, however, a main effect of species on alcohol dose consumed, such that meadow voles consumed a lower dose of alcohol than prairie voles (F(1,32) = 6.51; p = 0.012). Likewise, there was an effect of drinking condition, such that subjects drank more in the SD than the NSD condition (F(1,32) = 11.67; p = 0.0017), but there was no interaction (F(1,32) = 2.67; p = 0.11; Fig. 2B).

We further examined fluid volumes consumed to address the discrepancy between the species difference in the dose of alcohol consumed and the lack of species difference in alcohol preference. There was no difference in water consumption between species (F(1,32) = 1.38; p = 0.24), between drinking conditions (F(1,32) = 1.88; p = 0.18), nor an interaction (F(1,32) = 0.25; p = 0.62). There was no main effect of species on alcohol volume consumed (F(1,32) = 0.21; p = 0.65), but there was a main effect of drinking condition paralleling the difference in dose consumed (F(1,32) = 16.98; p = 0.0002). There was also an interaction between species and drinking condition (F(1,32) = 5.82; p = 0.022); however, post-hoc analysis revealed no difference between species during either SD or NSD conditions (Fig. 2C). Likewise, there was no species difference in the total volume of fluid consumed, (F(1,32) = 0.093; p = 0.76), but there was a main effect of drinking condition paralleling the difference in alcohol consumption (F(1,32) = 15.20; p = 0.0005). There was a trend for an interaction between drinking condition and species (F(1,32) = 4.04; p = 0.053; Fig. 2D).

**Figure 3:**
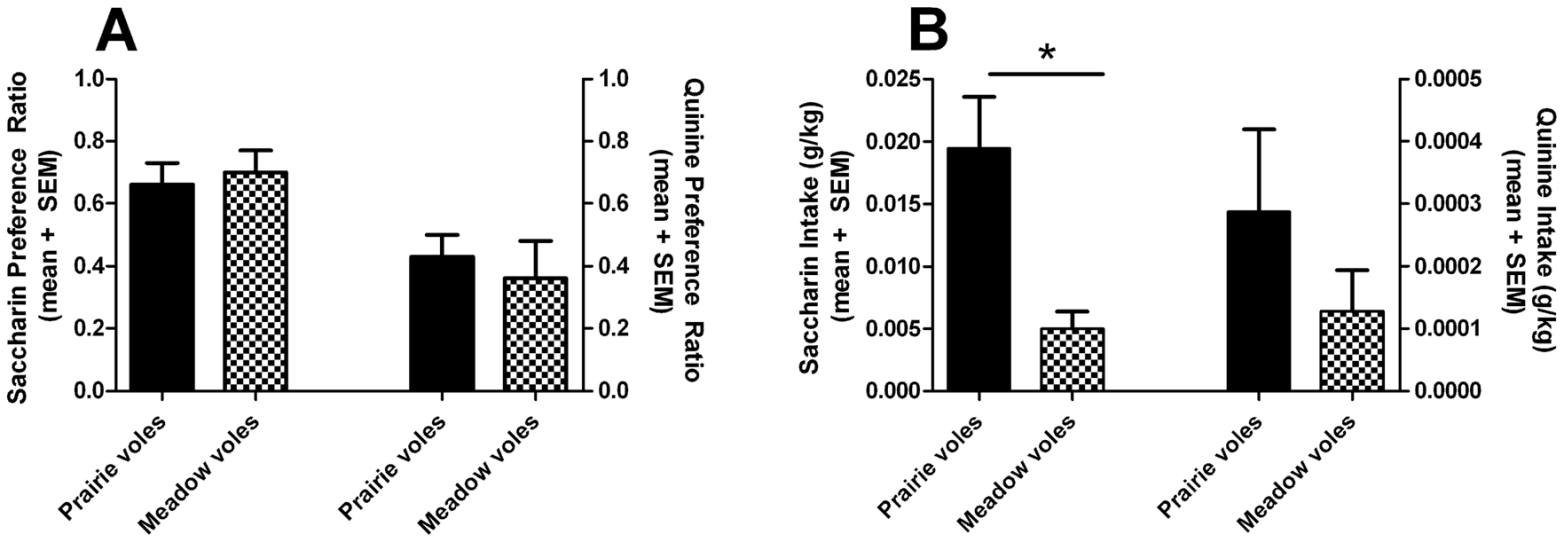
Consumption of tastants saccharin and quinine. There was no difference between species in tastant preference (A). There was a significant difference between species in tastant consumption, where meadow voles consumed less saccharin, but not quinine, than prairie voles (B). Values indicate group mean + standard error of the mean (SEM). * effect of species; p<0.05

There was no main effect of species on taste preference for saccharin (t = 0.37, df = 29, p = 0.71) or quinine (t = 0.54, df = 28, p = 0.59; Fig. 3A), but there was a significant effect of species on the amount of saccharin consumed per kg body weight (t = 2.39, df = 31, p = 0.023), where meadow voles consumed lower doses of saccharin, but not quinine (t = 0.81, df = 29, p = 0.42; Fig. 3B). The volume of saccharin consumed by meadow voles was significantly lower than that of prairie voles (Welch's t = 2.39, df = 29, p = 0.024).

**Figure 4:**
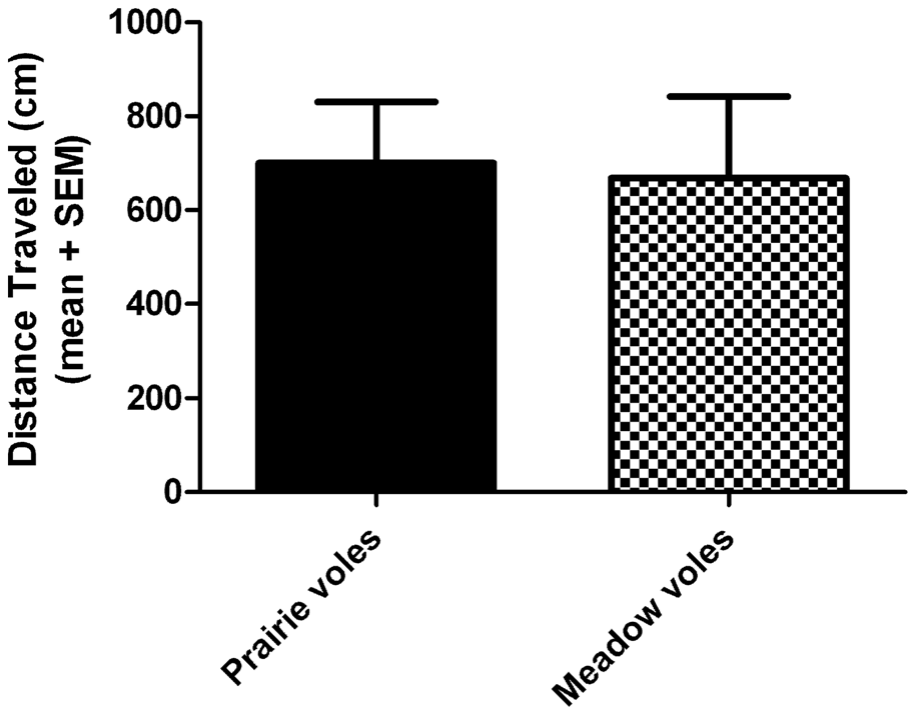
Locomotor activity during alcohol self-administration. There was no difference between species in the total distance traveled per day, averaged across all four days of 4-hour access. Values indicate group mean + standard error of the mean (SEM).

We also assessed locomotor activity. Averaged across all four days (four hours per day), there was no effect of species on the total distance moved within the drinking apparatus (t = 0.15, df = 22, p = 0.89; Fig. 4).

**Figure 5:**
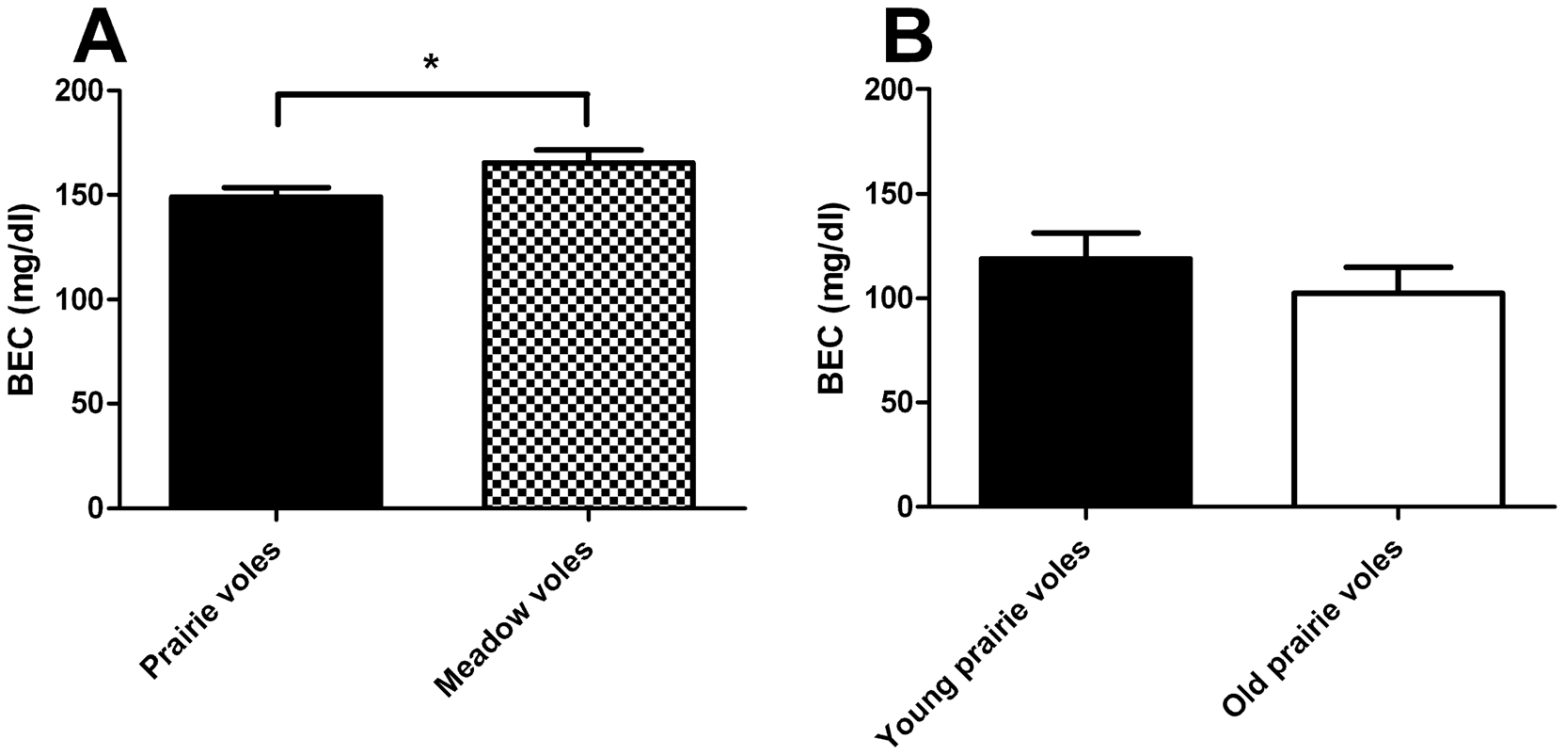
Blood ethanol concentrations (BECs) 90 minutes following a 2.5 g/kg intraperitoneal injection of ethanol. Meadow voles had significantly higher average BEC than prairie voles in Experiment 1 (A). There was no difference in average BEC of young and old prairie voles in Experiment 2 (B), age-matched to the prairie and meadow voles, respectively, from Experiment 1. Values indicate group mean + standard error of the mean (SEM). * effect of species; p<0.05

Despite consuming less alcohol per body weight than prairie voles (see Fig. 2B), meadow voles exhibited greater signs of intoxication, such as wobbly ambulation, sedation, and difficulty rearing up to the sipper. Therefore, we tested the rate of alcohol elimination in the two species as a proxy for identifying specific metabolic differences. Following injections of identical doses of alcohol relative to body weight (2.5 g/kg), meadow voles had a small (11%) but significant increase in BECs compared to prairie voles (t = 2.15, df = 31, p = 0.039; Fig. 5A).

**Figure 6:**
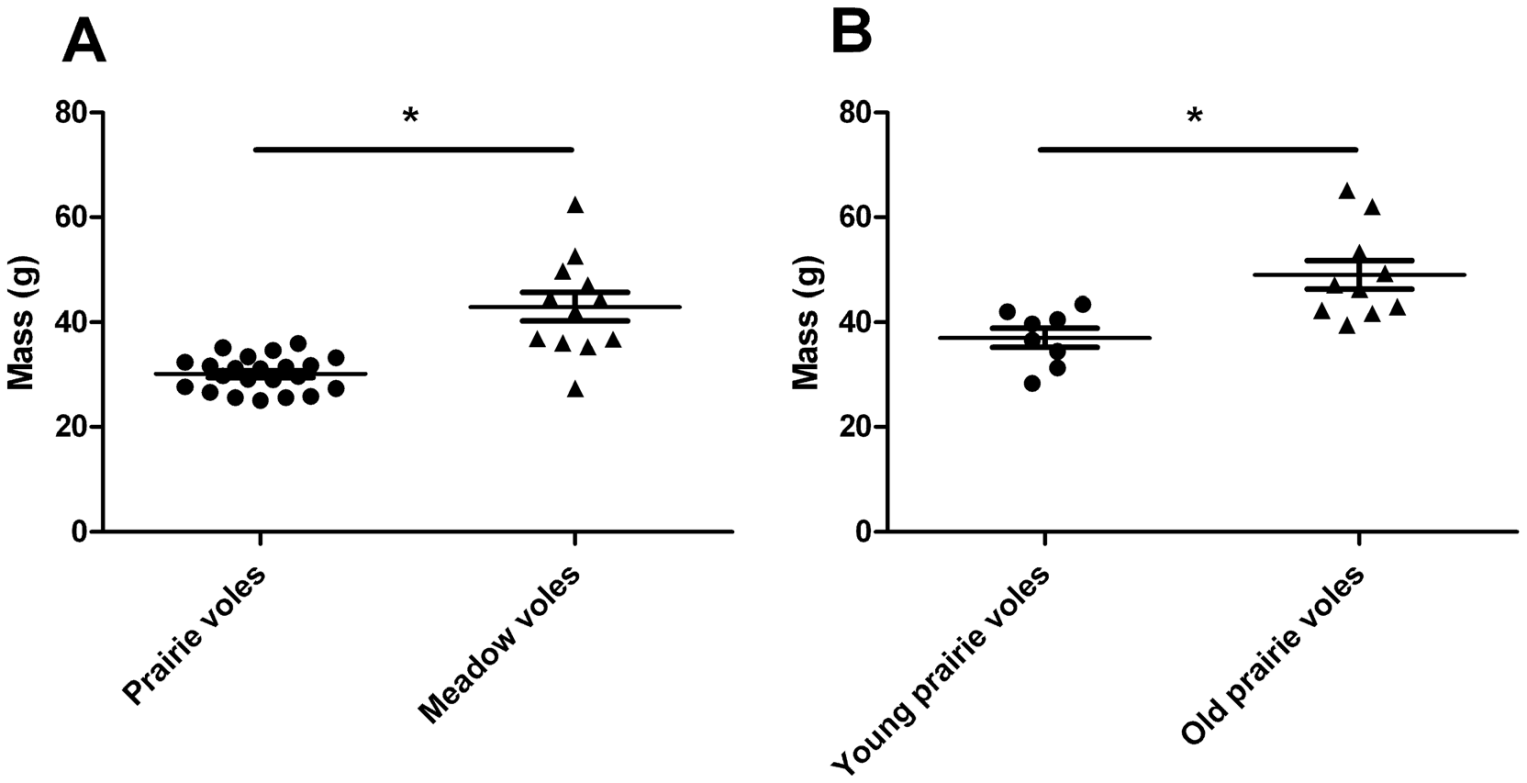
Body mass. Meadow voles had significantly greater body mass than prairie voles in Experiment 1 (A). In Experiment 2, older prairie voles age-matched to meadow voles from Experiment 1 had significantly greater body mass than younger prairie voles (B). Points indicate individual body mass; horizontal lines indicate mean ± SEM. * effect of group; p<0.005

**Figure 7:**
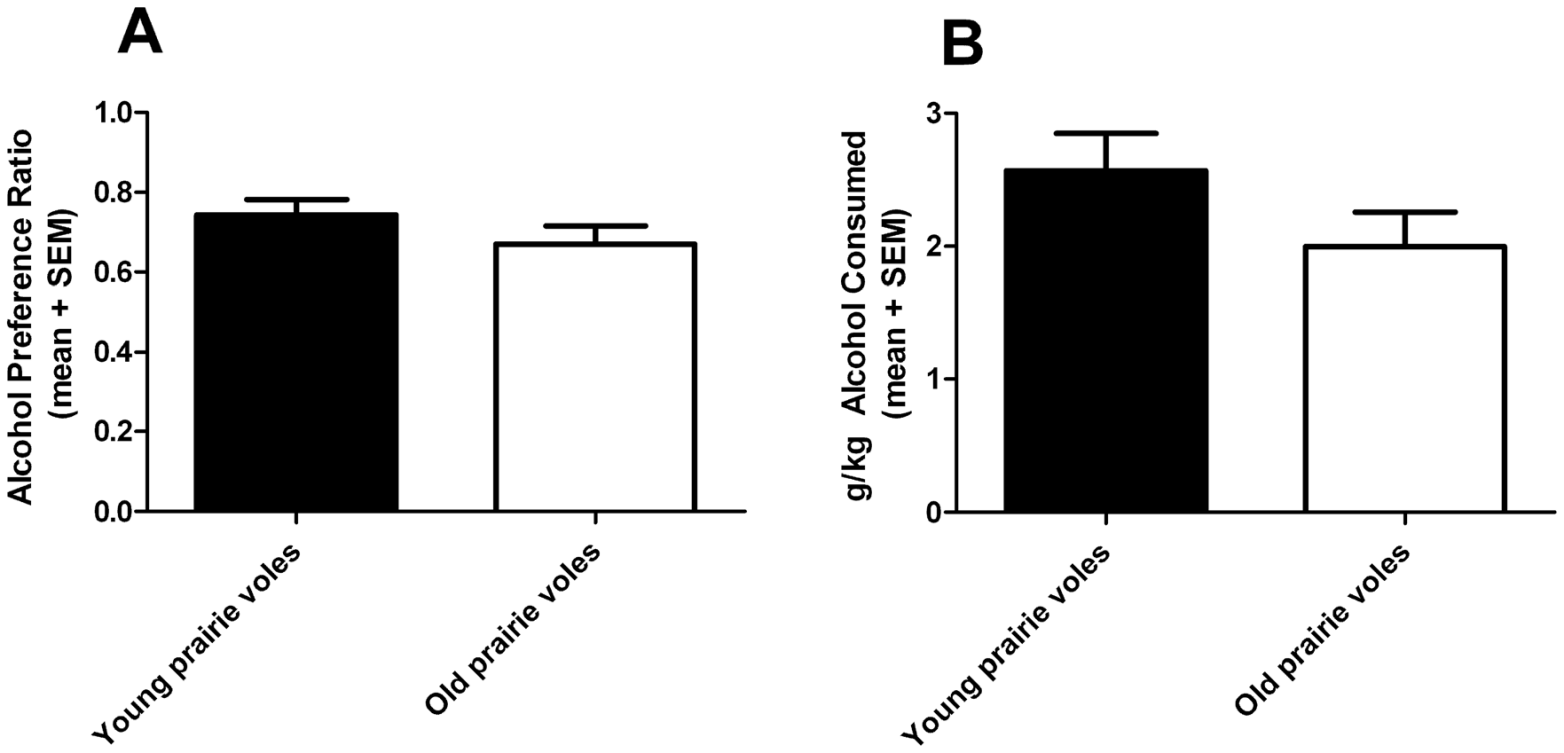
Alcohol drinking in young and old prairie voles. There was no significant difference in preference (A) or intake of alcohol (B) as a result of age or body mass. Values indicate group mean + standard error of the mean (SEM).

In Experiment 1, meadow voles were significantly older and weighed significantly more than the prairie voles (Welch's t = 4.58, df = 12, p = 0.0006; Fig. 6A), potentially confounding our species comparisons. Thus, in Experiment 2, we conducted a within-species comparison of young and old prairie voles. As expected, older prairie voles weighed more than young prairie voles (t = 3.57, df = 15, p = 0.0028; Fig. 6B). Despite the difference in age and weight, young and old prairie voles exhibited no differences in alcohol preference (t = 1.20, df = 16, p = 0.25; Fig. 7A) or alcohol dose consumed (t = 1.49, df = 16, p = 0.15; Fig. 7B). Moreover, there was no difference in BEC between young and old prairie voles (t = 9.12, df = 15, p = 0.38; Fig. 5B).

## Discussion

In this study we found that, in prairie voles, early life family structure had no impact on later life alcohol drinking. The most striking finding in this study is the species difference in alcohol consumption; given equal access to alcohol, meadow voles voluntarily consume lower doses of alcohol than prairie voles. This is consistent with our working hypothesis that the previously identified species differences in reward circuitry may lead to differences in the intake of alcohol. Complicating this hypothesis, however, is the unexpected finding that both vole species drank more alcohol in the social drinking (SD) condition than in the nonsocial drinking condition (NSD). Based on our working hypothesis, we would have expected meadow voles to behave more like mice or rats, which tend to drink less under social conditions (reviewed in [16]). Both findings, however, must be interpreted cautiously.

First, speculations regarding the mechanisms underlying the species differences in alcohol consumption must be tempered by our finding of species differences in BEC. Following a fixed dose of alcohol, meadow voles had a small but statistically significant increase in BECs in comparison to prairie voles (Fig. 5A). It is possible that meadow voles might not metabolize and eliminate alcohol as quickly. Such a difference in pharmacokinetics may contribute to the observed differences in self-administration (Fig. 2B). If meadow voles simply require less alcohol than prairie voles to reach the same pleasurable or intoxicating BEC level, then species comparisons of the rewarding or reinforcing effects of alcohol or of the relevant brain circuits may prove difficult to interpret. However, the absolute magnitude of the BEC disparity between species is relatively small (11%). Thus, it is unlikely that there are major differences in the ethanol metabolism pathway. Hence, the findings of this study do not preclude future studies of alcohol self-administration in meadow voles, but do make direct comparisons between these vole species more complicated.

In Experiment 1, the species difference in alcohol dose intake, but not preference, ethanol volume, or total volume of fluid intake, might also be explained by the differences in age and greater mass of the older meadow voles compared to the prairie voles. Our second experiment directly assessed the potential contribution of age and body mass on drinking and BECs by comparing young and old prairie voles; the findings indicated no effect of age or mass. Thus, the difference between prairie and meadow voles in alcohol intake and BEC is likely due to a real difference between the species, and is not due to the age or body weight differences. It should be noted that as the prairie voles in Experiment 2 were selected by age, their body mass is not accurately matched to the respective groups in Experiment 1; in fact, the young prairie voles' average body mass is slightly more similar to that of the meadow voles, while the older prairie voles have even greater mass. Thus direct comparisons between the two experiments should not be considered, but the results of Experiment 2 show that the difference in age and mass in prairie voles has no effect on alcohol intake or BEC.

The observed difference in saccharin dose and volume intake reinforces the idea that meadow voles will consume less of a rewarding substance compared to prairie voles, even while having the same preference. However, this appears not applicable to a non-rewarding substance, such as quinine. Alternatively, no differences may have been detected for quinine intake due to a floor effect, since very small volumes of this bitter substance were consumed by both species.

Interestingly, both prairie voles and meadow voles consumed more alcohol in the social drinking condition (SD) than the non-social drinking condition (NSD). While the prairie vole data are consistent with our previous studies of socially-facilitated alcohol drinking in prairie vole siblings or cagemates [15], obtaining similar findings with meadow voles was perhaps unexpected. It could be hypothesized that meadow voles would behave more like mice, another social but non-monogamous rodent species often used in alcohol drinking studies. In our laboratory, C57BL/6J mice show no evidence of the socially facilitated alcohol drinking that is exhibited by prairie voles (A.M.J. Anacker, M.R. Painter and A.E. Ryabinin, unpublished results). Instead of behaving like this other promiscuous species, we find that meadow voles do exhibit social facilitation of alcohol drinking similar to prairie voles, and in fact do so from the beginning of alcohol availability. The only other study to show social facilitation of alcohol drinking in a promiscuous species required seven weeks of alcohol consumption with older adult mice before the effect was seen [47].

The interpretation of this finding, however, benefits from a closer look at the social biology of this species. Although meadow voles are non-monogamous, they do form specific social attachments to siblings and cagemates [24], [25]. It may be that, while the neurobiological mechanisms for opposite-sex attachments are different in prairie and meadow voles [48], both species use similar mechanisms for same-sex affiliations, which may impact alcohol intake in the same way. Indeed, evidence from meadow voles suggests that oxytocin receptor levels in the lateral septum and central nucleus of the amygdala may play a role in the affiliative bond [48], and these regions have been implicated in alcohol use [49], [50], [51], [52]. Thus, both vole species may model effects of social interactions with peers increasing alcohol intake.

It should also be noted that the present methodology is not identical with our previous studies that demonstrated effects of social housing on alcohol drinking [15], since the NSD condition in the present study was achieved with non-adjacent drinking tubes and the animals were never fully isolated. Importantly, these results demonstrate that we are able to observe the peer influences on alcohol drinking across several different procedures with prairie voles.

In addition to species comparisons, we also assessed the influence of rearing condition. We have shown previously [42], [45] that both mothers and fathers spend similar large amounts of time on the nest with pups, and that in BP conditions the parents coordinate time away from the nest, leaving the pups unattended as little as possible. Thus, with no father present, under SM conditions the pups are exposed more frequently. While the father is actively involved in parenting, he typically does not lick and groom as much as the mother. However, under SM conditions, pups receive less total licking and grooming. Based on differences in adult social behavior of SM- and BP-reared prairie voles [42], we had hypothesized that SM-reared animals might consume more alcohol and be less sensitive to social facilitation. Contrary to this hypothesis, there was no difference between SM and BP prairie voles on any of the experimental measures. This indicates that, at least in this animal model of parenting, experiencing lower levels of normal parental care and paternal deprivation are not significant risk factors for greater alcohol use in adulthood. While this appears contrary to some of the literature regarding human alcohol intake [33], [34], [35], [36], [37], the literature is mixed. Some studies have found no effect of non-intact family structure on alcohol intake [38], [39], while others have found that apparent effects of family structure disappear when adjusted for other covariate factors, or are weaker than other mediating variables [40], [41]. In this biological system, without the influence of other confounding factors, prairie voles do not show any effect of parenting on alcohol drinking. This lends support to the idea that it may indeed be cultural or other environmental factors coincident with single parenting that lead to increased alcohol use in humans.

There is also conflicting evidence of the role of diminished parental care in alcohol use and abuse from other animal models (reviewed in [16]). Rodent models traditionally used to study alcohol intake do not exhibit bi-parental care, and thus early weaning or periods of maternal separation are typically used to model reduced parental care. This type of deprivation in mice and rats has variably lead to increased drinking [53], [54], [55], conditional effects [56], no effect [57], and even decreased alcohol intake [58]. While it may be argued that these models are valuable for their construct and predictive validity (reviewed in [59]), the prairie vole model of bi-parental care appears to offer better face validity, because in these species both the mother and father play an active role in parenting as is seen in most human families. In the rhesus macaque, non-traditional parenting (“peer-reared” as opposed to traditional “mother-reared”) leads to greater alcohol consumption later in life [60], [61]. However, as in mice and rats, the traditional mother rearing among macaques does not directly parallel the typical bi-parental rearing common to humans and prairie voles. In addition, the peer-rearing conditions are quite different than the “non-intact” family structures of humans and the SM prairie voles in this study. In short, extreme differences in parenting may be required to influence the alcohol consumption of offspring later in life.

It is important to note that the laboratory SM and BP comparison captures only a fragment of the complex family structure of prairie voles. In the wild, these effects may be exaggerated since there is a wide range of family unit types: group housing is common and more likely to result in litters receiving constant care, while mothers rearing pups alone in the wild would have greater demands on their time to find food, and leave pups exposed more often [62], [63], [64]. This diversity is strikingly similar to human family units, which can range from single mother to bi-parental, to communal or extended family groups, where the father typically actively participates in parenting. Nevertheless, while there are many parallels between humans and prairie voles in their social bonding and underlying neurobiology, social alcohol intake, and dual-parenting styles, the construct validity (i.e., similarity in underlying causes of these behaviors) has yet to be shown.

In conclusion, prairie voles show no effect of SM-rearing when compared to typical BP-rearing on measures of alcohol drinking, indicating that the social biological effects of non-intact family structure in this animal model might not be sufficient to explain differences observed in humans from single-parent homes. Instead, family structure may be moderated by other environmental and cultural effects that influence alcohol drinking. Additionally, meadow voles self-administer significantly less alcohol than prairie voles, while also achieving a higher BEC from a similar dose, indicating that future species comparisons require caution and a more detailed analysis of elimination rates. Meadow voles may still provide an interesting model system for alcohol drinking behaviors in their own right, as they demonstrate a preference for alcohol, visible signs of intoxication, and social facilitation of alcohol drinking. Both vole species demonstrate characteristic social behaviors that have established them as unique models of human social behavior. This study shows that, similar to socially monogamous prairie voles, non-monogamous meadow voles voluntarily self-administer alcohol and their intake can be influenced by the social environment, quite differently than what has been demonstrated in traditional laboratory models. Future studies may examine interactions between species-specific social behaviors and alcohol drinking in each of these vole species.
